# Favism: A Case Report

**DOI:** 10.7759/cureus.23269

**Published:** 2022-03-17

**Authors:** Andreia Diegues, Pedro Simões, Tiago Ceriz, Ana Rita Lopes, Elisa Tomé

**Affiliations:** 1 Internal Medicine Department, Unidade local de Saúde do Nordeste, Bragança, PRT; 2 Internal Medicine Department, Unidade Local de Saúde do Nordeste, Bragança, PRT; 3 Internal Medicine Department, Centro Hospitalar Médio Ave, Santo Tirso, PRT

**Keywords:** glucose-6-phosphate dehydrogenase deficiency, fava beans, hemolysis, hemolytic anemia, favism

## Abstract

Favism is an acute hemolytic syndrome that occurs in patients with glucose-6-phosphate dehydrogenase (G6PD) deficiency following the ingestion of fava beans. Diagnosis can be challenging because the severity of hemolytic anemia varies among this patient population. Furthermore, the severity of hemolytic episodes can vary in the same patient. The diagnosis of G6PD deficiency and patient education pertaining to safe and unsafe medications and foods are crucial to prevent the reoccurrence of hemolytic episodes. Here, we report the case of a man admitted to our hospital with an acute hemolytic episode. At the time of admission, we were unaware that he had ingested fava beans and only discovered that he had G6PD deficiency while performing complementary studies during the hemolytic crisis to determine its etiology.

## Introduction

Glucose-6-phosphate dehydrogenase (G6PD) deficiency is the most common enzymatic disorder of red blood cells (RBCs). This inherited chromosomal disorder was discovered in 1956. G6PD catalyzes the first reaction of the hexose monophosphate shunt, generating NADPH. This pathway is ultimately responsible for the reduction of reactive oxidative species, thereby protecting RBCs from oxidative stress [1,2]. G6PD deficiency is widely distributed across populations but is highly prevalent in Africa, Southern Europe, the Middle East, Southeast Asia, and Oceania [3]. The clinical presentation of G6PD deficiency includes different manifestations across a wide spectrum of severity. The World Health Organization has classified G6PD deficiency as class I-IV according to the magnitude of the enzyme deficiency. Patients in class II have a severe enzyme deficiency, where the G6PD activity is <10% of the normal value. Class II patients experience intermittent hemolytic episodes, typically after exposure to substances that are a source of oxidant stress, such as fava beans (as in this case) or oxidant medications. G6PD deficiency can also be classified according to mutations in the G6PD gene that exist within specific ethnic groups, such as Mediterranean-type G6PD deficiency, which is a class II deficiency [4,5].

Because G6PD deficiency is an X-linked recessive disorder, the main clinical manifestations are observed in hemizygous males, and most females are unaffected carriers [1,3]. The most frequent manifestations are neonatal jaundice and acute hemolytic anemia, which typically appear 2-4 days after exposure to a trigger, such as certain medications, toxins, or agents of infection [6,7].

Favism refers to an acute hemolytic episode in G6PD-deficient individuals that occurs after ingesting fava beans [2]. It is the most common cause of acute hemolytic anemia in patients with G6PD deficiency. The first unequivocal reference to favism appeared in an article published by Mira Franco in 1843 in Portugal in the Revista Universal Lisbonense. In 1894, the term was introduced by Montano at the Ninth International Medical Congress in Rome [3,8].

Here, we report a male patient with G6PD deficiency who presented with severe hemolytic anemia and low levels of G6PD during the hemolytic crisis. Furthermore, we emphasize that the intensity of hemolytic episodes can vary in the same patient [9].

## Case presentation

A 67-year-old, Caucasian man was admitted to the emergency department complaining of generalized abdominal pain over the past few days that was associated with nausea, anorexia, brown urine, and yellow sclerae and skin. He also developed a fever on the day prior to admission. His medical history revealed arterial hypertension and chronic hepatic disease with esophageal varices that we suspected were probably of alcoholic etiology. He also experienced an intermittent episode of jaundice one year ago, where the cause was undetermined.

Upon physical examination, the patient presented with a fever (38.2 ºC), malar telangiectasia, jaundice, and generalized abdominal pain that was worse in the right hypochondrium and without organomegaly. No cardiovascular or respiratory system abnormalities were noted. The results of laboratory tests are presented in Table 1. The level of hemoglobin (Hb) was low, and liver enzymes, total bilirubin, and creatine kinase were elevated. The peripheral blood smear showed macrocytosis and leukocytosis. Urinalysis showed bilirubinuria and hemoglobinuria.

**Table 1:** Laboratory test results on admission. CK, creatine kinase; GOT, glutamic oxaloacetic transaminase; INR, international normalized ratio; LDH, lactate dehydrogenase

Parameter	Result	Normal range
Hemoglobin (g/dL)	9.2	14.0-17.5
Total leucocyte count (x10^9^/L)	19.02	4.4-11.3
Differential leucocyte count (%)		
Neutrophils	70.8	50-70
Lymphocytes	15.7	25-40
Monocytes	13.1	2-8
Eosinophils	0.1	1-4
Platelet count (x10^9^/L)	225	150-450
LDH (U/L)	1708	<250
Total bilirubin (mg/dL)	10.75	0.3-1.2
Direct bilirubin (mg/dL)	0.81	<0.2
GOT (U/L)	96	<31
CK (U/L)	432	<271
INR	1.27	-
Fibrinogen (mg/dL)	255	200-400

Abdominal computed tomography revealed a normal-sized liver with no focal lesions, patency of the portal vein, the absence of intra- or extrahepatic bile duct dilatations, no vesicular lithiasis, and no abnormal changes in the spleen or other organs.

Because the clinical and laboratory findings suggested acute hemolysis, we performed further tests to investigate the underlying etiology (Table 2). The complementary laboratory workup revealed an elevated reticulocyte count, low levels of haptoglobin, and folic acid deficiency. The Coombs test was negative, and thyroid function was normal.

**Table 2:** Results of the complementary lab workup performed to determine the underlying etiology of acute hemolytic anemia in our patient. TSH, thyroid-stimulating hormone; T4, thyroxine; HBV, hepatitis B virus; HCV, hepatitis C virus; HIV, human immunodeficiency virus

Parameter	Result	Normal range
Reticulocyte count (%)	3.6	0.5-1.5
Haptoglobin (mg/dL)	3	30-200
Coombs test	Negative	
Folic acid (ng/mL)	2.1	3.1-20.5
Ferritin (ng/mL)	8 652	21.81-274.66
Transferrin saturation (%)	91.2	20-50
Iron (µg/dL)	165	70-180
TSH (µUi/mL)	0.87	0.35-4.94
Free T4 (ng/dL)	0.93	0.70-1.48
HIV	Negative	
HBV	Negative	
HCV	Negative	

**Figure 1:**
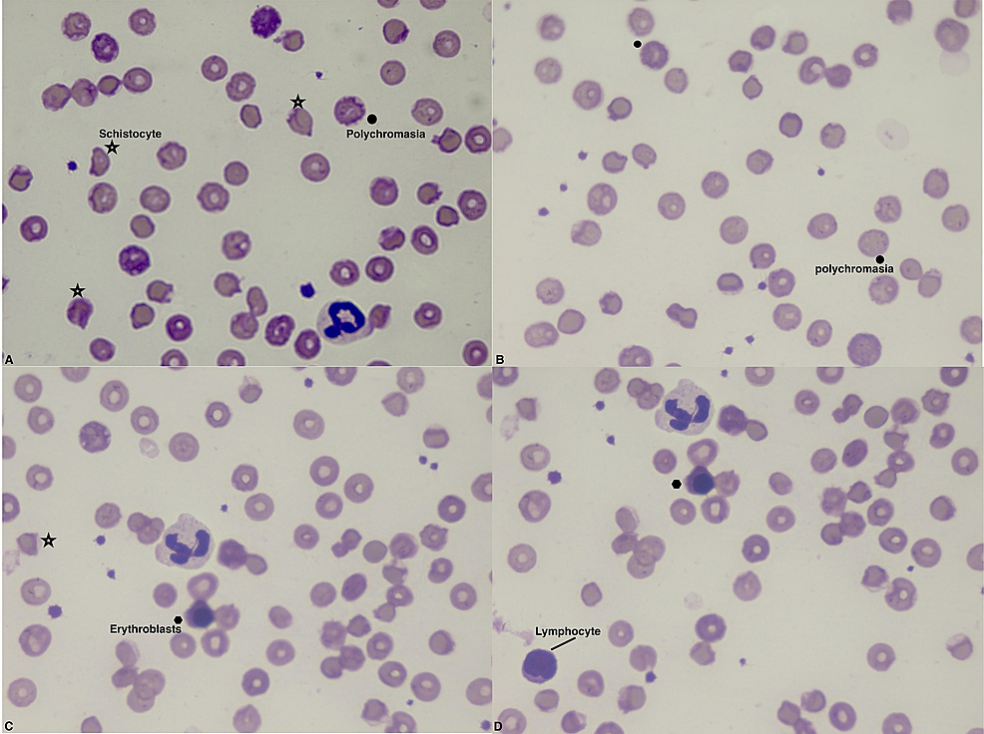
Peripheral blood smear at admission. The smear was stained with May–Grünwald–Giemsa solution. There is marked anisocytosis and poikilocytosis, which is unspecific. Polychromasia is present, which is typically observed in blood disorders that result in the premature release of RBCs from the bone marrow. Schistocytes (RBC fragments indicating hemolysis) are also present. Erythroblasts with off-center nuclei containing dense chromatin can be seen; these are typical of severe anemia and a characteristic finding of hemolytic disease. Neutrophil hypersegmentation is visible in panels A, C, and D, and lymphocyte is present in panel D. Star = schistocyte, black dot = polychromasia.

The peripheral blood smear showed polychromasia and anisopoikilocytosis, including schistocytes, erythroblasts, and macrocytes (Figures 1A-1D). Taken together, the results of the initial and complementary workup were consistent with a diagnosis of hemolytic anemia and leukocytosis with neutrophilia and neutrophil hypersegmentation.

During the first few days of hospitalization, the patient’s Hb dropped to 6.8 g/dL and renal function deteriorated. An increased urea concentration of 116 mg/dL (normal range: 17-43 mg/dL) and creatinine of 1.4 mg/dL and than 1.6 and 1.8 mg/dL (normal range: 0.91-1.4 mg/dL) was observed, developed into acute renal failure.

After treating the patient with fluid therapy and two units of RBCs, his Hb increased to 8.2 g/dL. He also received folic acid supplementation. Urine output returned to normal levels, and renal function started improving. All samples sent for microbial culture were negative. On the fifth day of hospitalization, he showed progressive clinical improvement, with decreased jaundice and improvement in urine color as well as improved laboratory test results. This improvement in our patient’s condition confirmed our suspicion of a hemolytic crisis. Subsequently, the patient′s general and renal condition recovered completely.

Further investigations revealed an ill-clarified picture of jaundice, without hematological disease, namely, jaundice or anemia, in the family. The patient had not recently taken any medication or received blood transfusions. There were no current or recent localized signs or symptoms of any infection, and he had never traveled abroad. On further questioning, the patient remembered that he had ingested fava beans (boiled fava beans and fava bean soup) approximately three days before the onset of his symptoms. Coincidentally, he had also ingested them a year ago when he experienced his previous episode of jaundice. These important details regarding fava bean ingestion suggested that the hemolytic crisis had been triggered by G6PD deficiency. Thus, we measured the enzymatic activity of G6PD. The resultant value was 9.2 U/g Hb (normal range: 10.0-14.0 U/g Hb), indicating a moderate degree of enzyme deficiency, which confirmed the diagnosis of G6PD deficiency.

The patient has not eaten fava beans since this episode and is careful not to take any drugs that could trigger another hemolytic episode. He has not experienced further hemolytic episodes to date.

## Discussion

G6PD deficiency must be considered in the differential diagnosis of intra- and extravascular hemolysis. A latent or asymptomatic G6PD deficiency may only show clinical manifestations under the appropriate conditions. In this case, the trigger event was the ingestion of fava beans [4].

Detecting G6PD activity during a hemolytic crisis can be problematic, and activity within the normal range cannot rule out the diagnosis because older RBCs, which have less enzymatic activity, are the first to hemolyze, while the younger RBCs, with almost normal enzymatic activity, remain in circulation. In this case, a G6PD deficiency was detected during the hemolytic crisis, supporting the diagnosis of class II, probably a Mediterranean-type G6PD deficiency [10]. In most cases, the hemolytic crisis is self-limiting once exposure to the trigger is stopped, as occurred in our case [3]. Depending on the number of RBCs that have hemolyzed, it takes approximately 6 days for the Hb concentration and hyperbilirubinemia to improve and 3-6 weeks to return to normal. The cornerstone of the management of this disorder is avoiding and removing triggers of oxidative stress in RBCs. In particularly severe cases, immediate hydration and the administration of compatible blood transfusions are necessary. Although G6PD deficiency has a good prognosis, it can sometimes lead to organ failure. As seen in this case study, acute renal failure is an important complication of severe hemolysis that is probably induced by hemoglobinuria, which leads to acute tubular necrosis [1].

Various theories have been proposed regarding the mechanism of hemolysis in favism. The most favored hypothesis is that favism is caused by two ꞵ-glycosides, vicine and convicine, that contain the pyrimidines divicine and isouramil. These produce free radicals during auto-oxidation, leading to hemolysis in people with G6PD deficiency. The blood smear shows polychromasia, anisocytosis, and poikilocytosis, which are all features of acute oxidant-induced hemolysis, as was described in this case. Interestingly, G6PD-deficient individuals do not develop favism every time they consume fava beans, and the reason for this is unclear. In our case, the beans were consumed in large amounts over three consecutive days and were eaten as both boiled beans alone and in soup, and this may have a bearing on the occurrence of hemolysis. However, in the previous episode one year earlier, the patient only consumed one small meal of fava beans and rice. This suggests that the quality and quantity of fava beans consumed influence the occurrence of a hemolytic episode [1,3,11].

## Conclusions

Although the Mediterranean-type G6PD deficiency is common and usually not difficult to diagnose, we have reported this case because favism can be encountered in daily clinical practice. G6PD deficiency should be suspected in cases of Coombs-negative hemolytic anemia where the patient has ingested fava beans or been exposed to oxidant medications. Furthermore, our case shows that hemolytic events due to G6PD deficiency could present with different degrees of severity in the same patient.

Importantly, hemolytic anemia in G6PD-deficient individuals can be triggered by substances other than fava beans, including a wide range of drugs, industrial chemicals, and alcohol. Thus, it is imperative to identify this mutation to prevent other episodes.
